# Supplementary material for: Progressive divisions of multipotent neural progenitors generate late-born chandelier cells in the neocortex
Source: Nat Commun. 2018 Nov 2;9:4595. doi: 10.1038/s41467-018-07055-7 (PMC6214958; doi:10.1038/s41467-018-07055-7)
Supplement: Supplementary file 1 — Supplementary Information [file 41467_2018_7055_MOESM1_ESM.pdf]

Progressive divisions of multipotent neural progenitors generate late-born chandelier cells in the neocortex

Sultan et al.

Supplementary Information

Supplementary Figures 1-12 and Figure Lengends

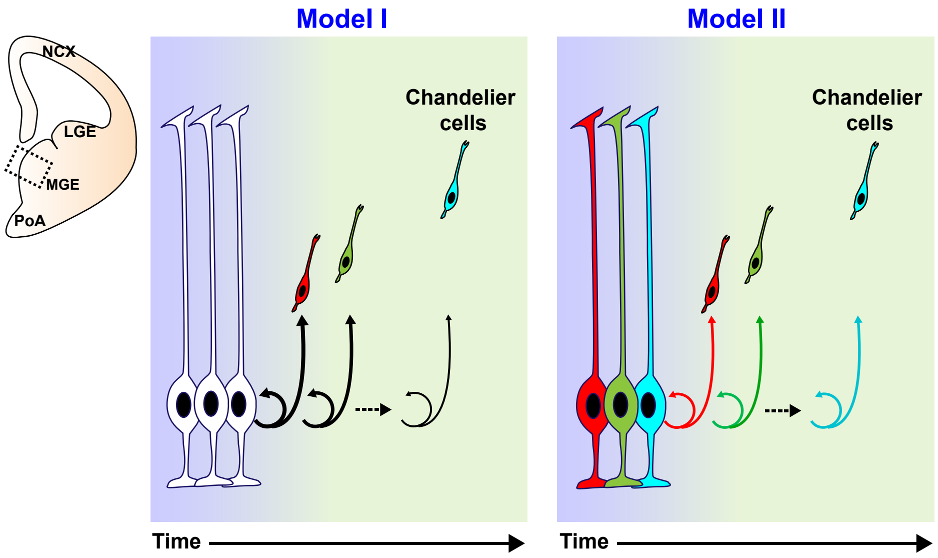

Sultan et al., Supplementary Figure 1

Supplementary Figure 1: Schematic of two possible models of NKX2.1^{+} MGE/PoA progenitor behavior underlying neocortical interneuron diversity. Model I depicts a common pool of multipotent progenitors that divide to produce distinct neocortical interneurons in a temporal, progressive specification manner. Model II depicts the existence of multiple pools of fate-restricted progenitors that divide at different times to produce distinct neocortical interneurons.

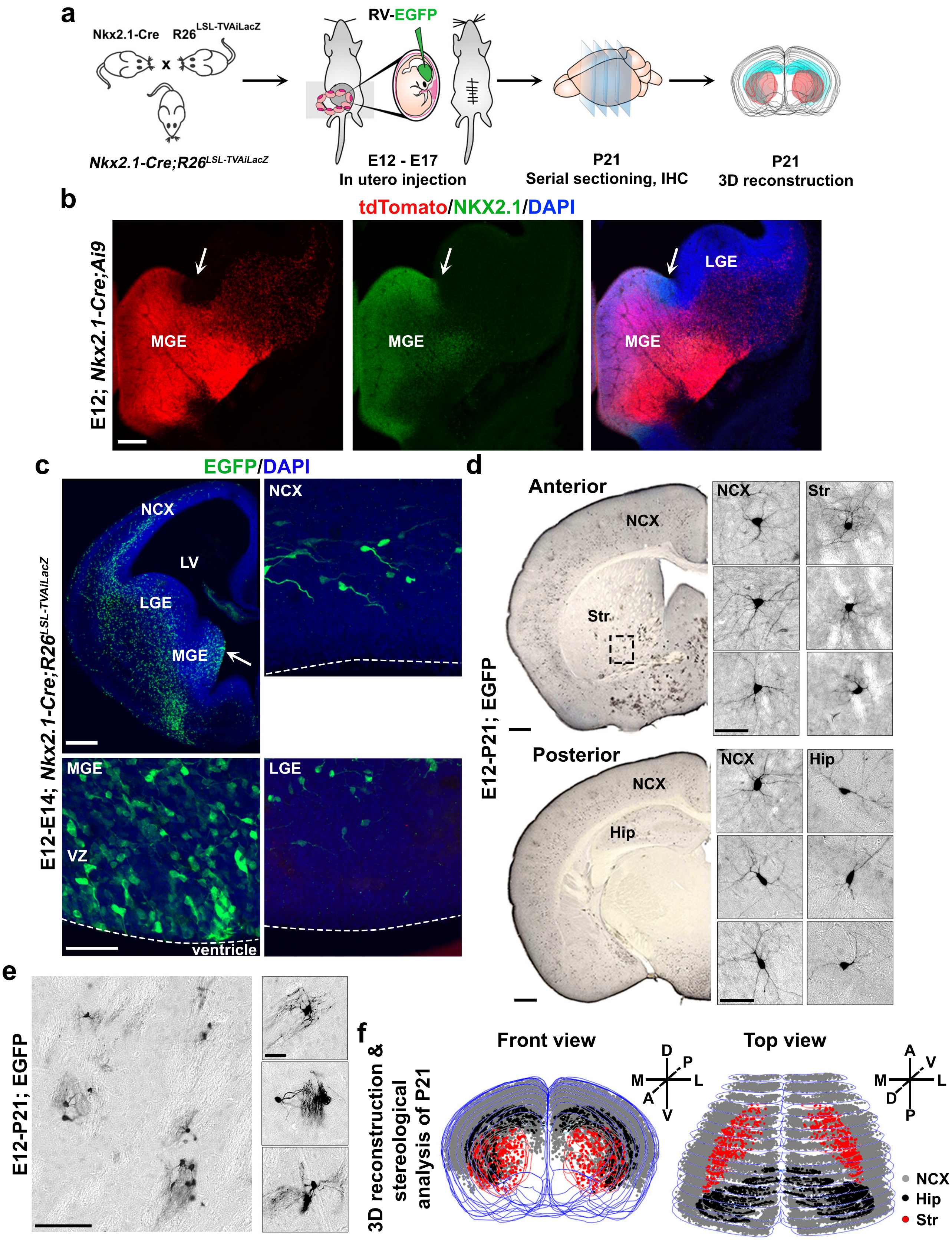

Sultan et al., Supplementary Figure 2

Supplementary Figure 2: Selective labeling of dividing NKX2.1^{+} MGE/PoA RGPs and their interneuron output in the neocortex. (a) Schematic of the experimental design. **(b)** Images of E12 *Nkx2.1-Cre;Ai9-tdtomato* mouse brain section stained for tdTomato (red), NKX2.1 (green), and counterstained with DAPI (blue). Arrow indicates the dorsal most edge of the MGE that lacks *Cre*-recombinase activity. MGE, medial ganglionic eminence; LGE, lateral ganglionic eminence. Scale bar: 150 µm. **(c)** Images of E14 *Nkx2.1-Cre;R26*$^{LSL\text{-}TVAiLacZ}$ mouse brain injected with RCAS-EGFP retrovirus at E12, and stained for EGFP (green) and counterstained with DAPI (blue). Higher magnification images of the VZ of the MGE, LGE, or NCX (neocortex) are shown to the bottom and right. The arrowhead indicates the specificity of the labeling of cells in the VZ of the MGE, but not the LGE or NCX. The broken lines indicate the VZ surface. LV, lateral ventricle. Scale bars: 250 µm and 50 µm. **(d)** Images of the anterior and posterior regions of P21 brain received *in utero* retrovirus injection at E12 and stained for EGFP (black). High magnification images of EGFP-expressing interneurons in the neocortex are shown to the right. Broken square indicates EGFP-expressing glial cells found in the subcortical region only. NCX, neocortex; Str, striatum; Hip, hippocampus; Scale bar: 500 µm and 100 µm. **(e)** Images of EGFP-expressing glial cells in the Striatum (Str, broken square in **c**). Scale bars: 100 µm and 15 µm. **(f)** 3D reconstruction images of P21 brain received in utero retrovirus injection at E12. Blue, black, and red lines represent the contours of the whole brain, Hip, and Str, respectively. Grey, black, and red dots represent the cell bodies of EGFP-expressing interneurons in the NCX, Hip, and Str, respectively. D, dorsal; V, ventral; A, anterior; P, posterior.

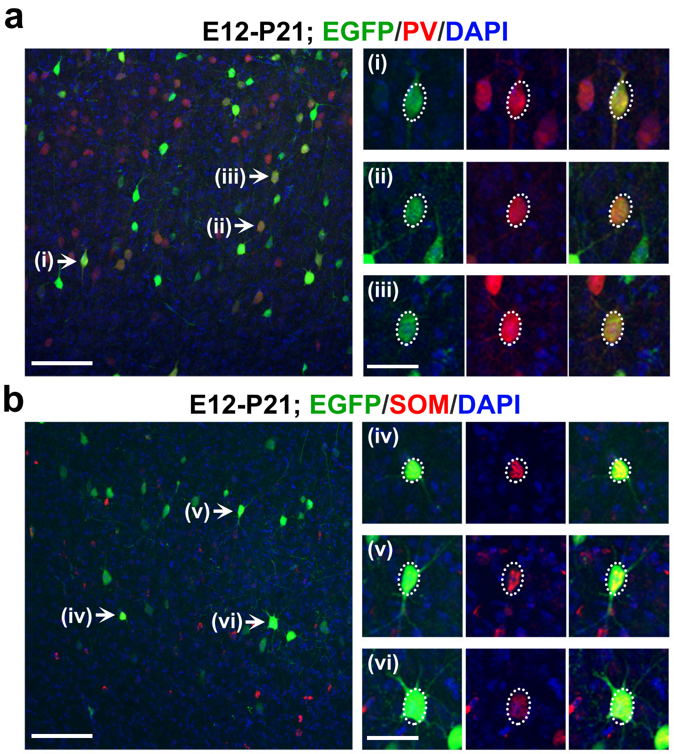

Sultan et al., Supplementary Figure 3

Supplementary Figure 3: Labeled NKX2.1$^+$ MGE/PoA RGPs produce characteristic neocortical interneurons. (a, b) Images of P21 neocortices that received *in utero* EGFP-expressing retrovirus injection at E12 were stained for EGFP (green), parvalbumin (PV, red, a) or somatostatin (SOM, red, b), and counterstained with DAPI (blue). High magnification images of individual EGFP-expressing cells (arrows) are shown to the right. Scale bars: 100 μm and 25 μm.

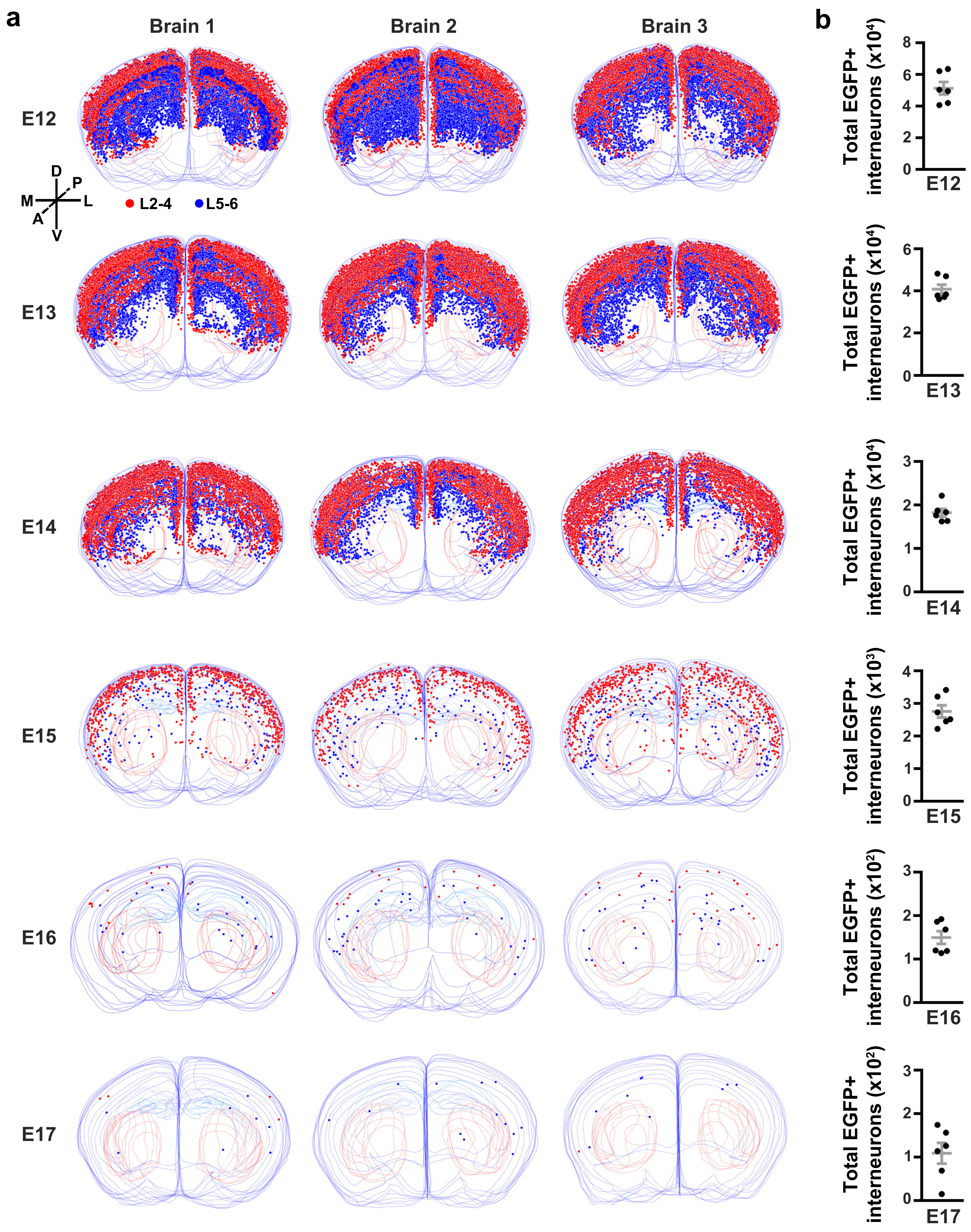

Sultan et al., Supplementary Figure 4

Supplementary Figure 4: Consistent labeling of neocortical interneuron progeny of dividing NKX2.1^{+} MGE/PoA RGPs by *in utero* retrovirus injection. (a) 3D reconstruction images of P21 brains that received *in utero* retrovirus injection at E12-E17. Blue lines represent the contours of the whole brain. Red and blue dots represent the cell bodies of EGFP-expressing interneurons in layers 2-4 and 5/6 of the neocortex, respectively. D, dorsal; V, ventral; A, anterior; P, posterior. **(b)** Quantification of the number of EGFP-expressing interneurons per hemisphere of the neocortex (n=6 hemispheres).

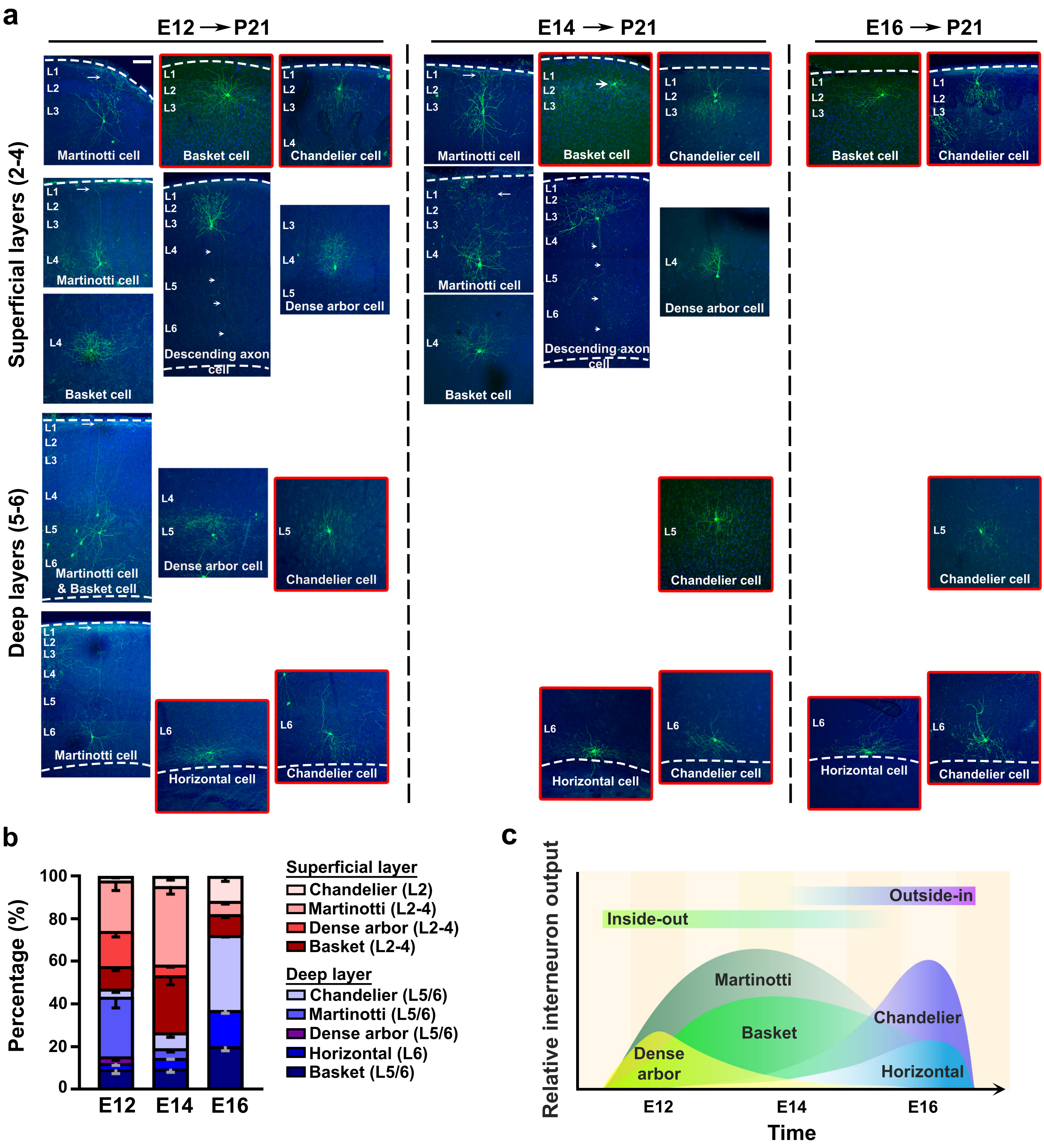

Sultan et al., Supplementary Figure 5

Supplementary Figure 5: Different subtypes of neocortical interneurons are progressively generated by dividing NKX2.1$^+$ MGE/PoA RGPs at different embryonic stages. (a) Representative images of EGFP-expressing interneurons (green) in the P21 somatosensory cortex labeled by intraventricular retrovirus injection at E12, E14, or E16, and stained with DAPI (blue). Broken lines indicate the pial surface or the white matter. The large arrows indicate the layer 1 projecting axons of Martinotti cells and the small arrows indicate the descending axons of descending axon cells. Red line squares indicate the cells that are labeled at all three embryonic stages. L, layer. Scale bar: 100 μm. **(b)** Quantification of the percentage of EGFP-expressing cells identified as Martinotti, dense arbor, basket, horizontal, or chandelier cells in the deep and superficial layers of the neocortex. Data are presented as mean ± SEM (n=3 hemispheres per embryonic stage). **(c)** Schematic representation of a progressive program in generating different subtypes of neocortical interneurons by NKX2.1$^+$ RGPs in the MGE/PoA.

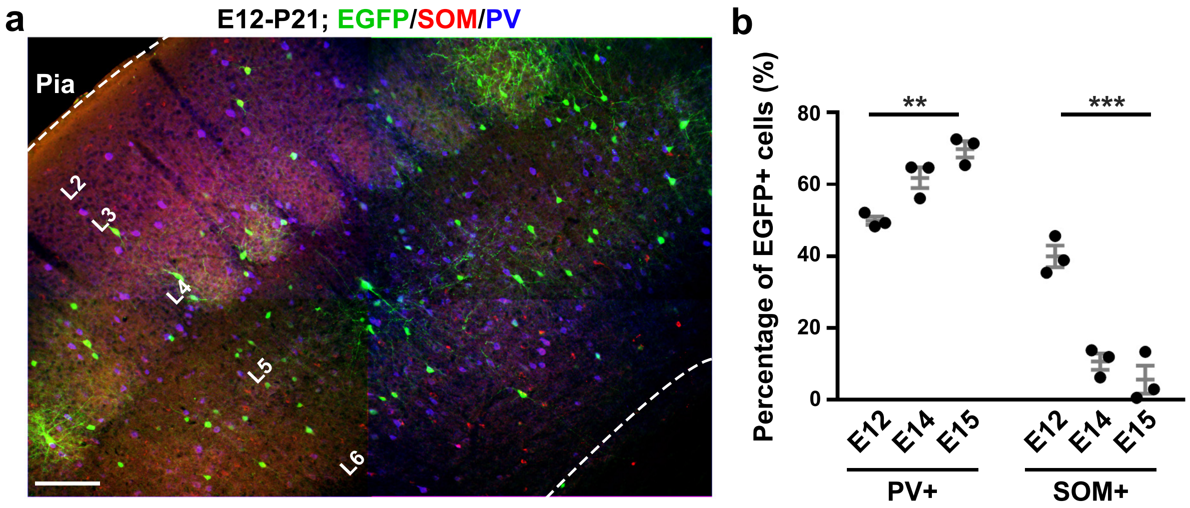

Sultan et al., Supplementary Figure 6

Supplementary Figure 6: Progressive production of PV^+ and SOM^+ neocortical interneurons by dividing $NKX2.1^+$ MGE/PoA RGPs. (a) Image of the somatosensory cortex of a P21 brain that received *in utero* EGFP-expressing retrovirus injection at E12, stained for EGFP (green), PV (blue), and SOM (red). Broken lines represent the pial surface and white matter boundaries. Scale bar: 250 µm. **(b)** Quantification of the percentage of EGFP-labeled cells expressing PV or SOM in the P21 brains injected with EGFP-expressing retrovirus at E12, E14, or E15. Grey lines represent mean ± SEM. Black dots represent individual brains (n=3 per time point). ** P=0.002; *** P=0.0005 (one-way ANOVA).

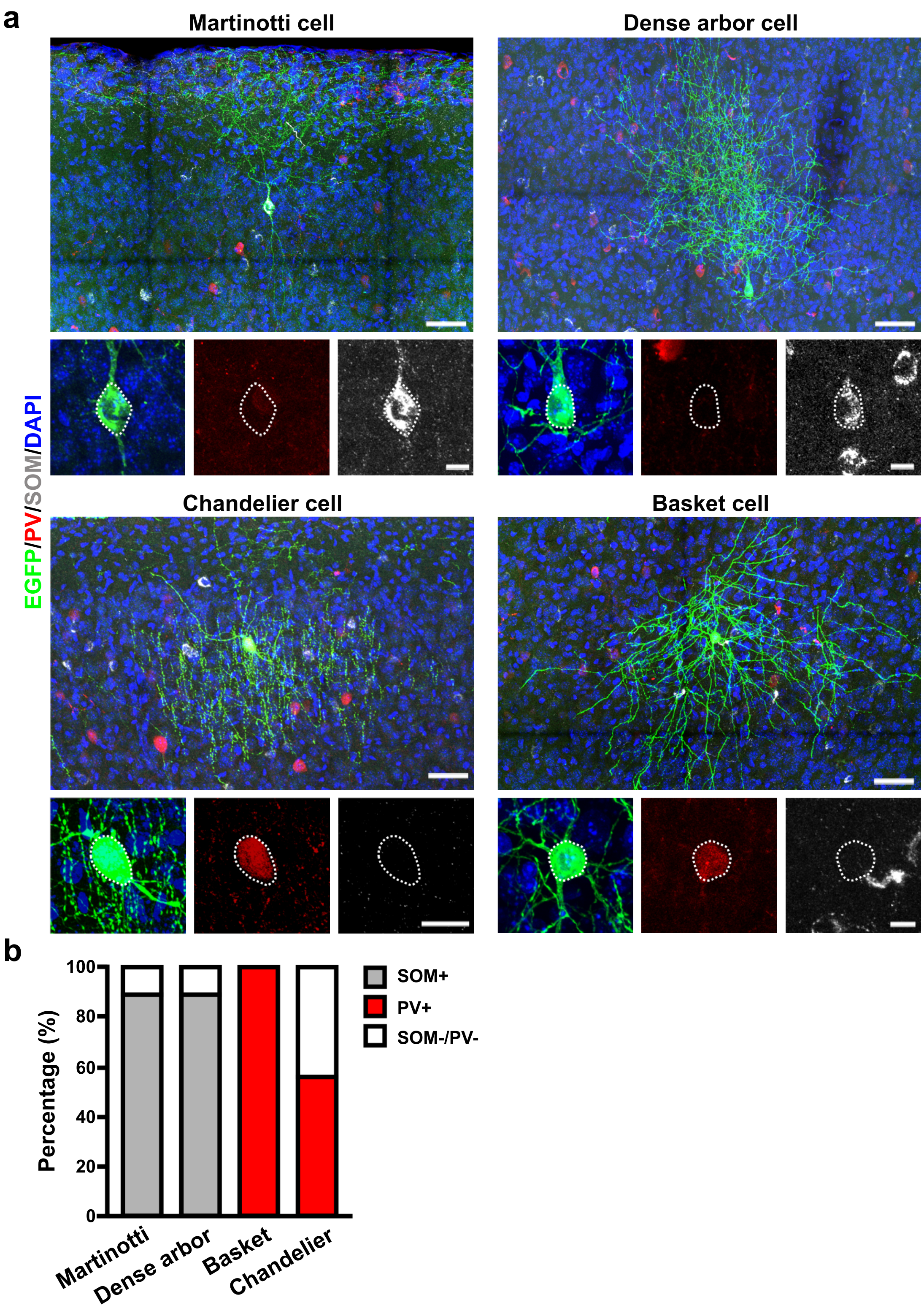

Sultan et al., Supplementary Figure 7

Supplementary Figure 7: Morphologically representative neocortical interneurons express PV or SOM. (a) Images of morphologically representative neocortical interneurons stained for PV or SOM. Scale bars: 50 and 20 μm. **(b)** Quantification of the percentage of Martinotti (n=82), dense arbor (n=18), basket (n=35), and chancelier (n=16) cells that are PV^+, SOM^+, or not stained (SOM^-/PV^-).

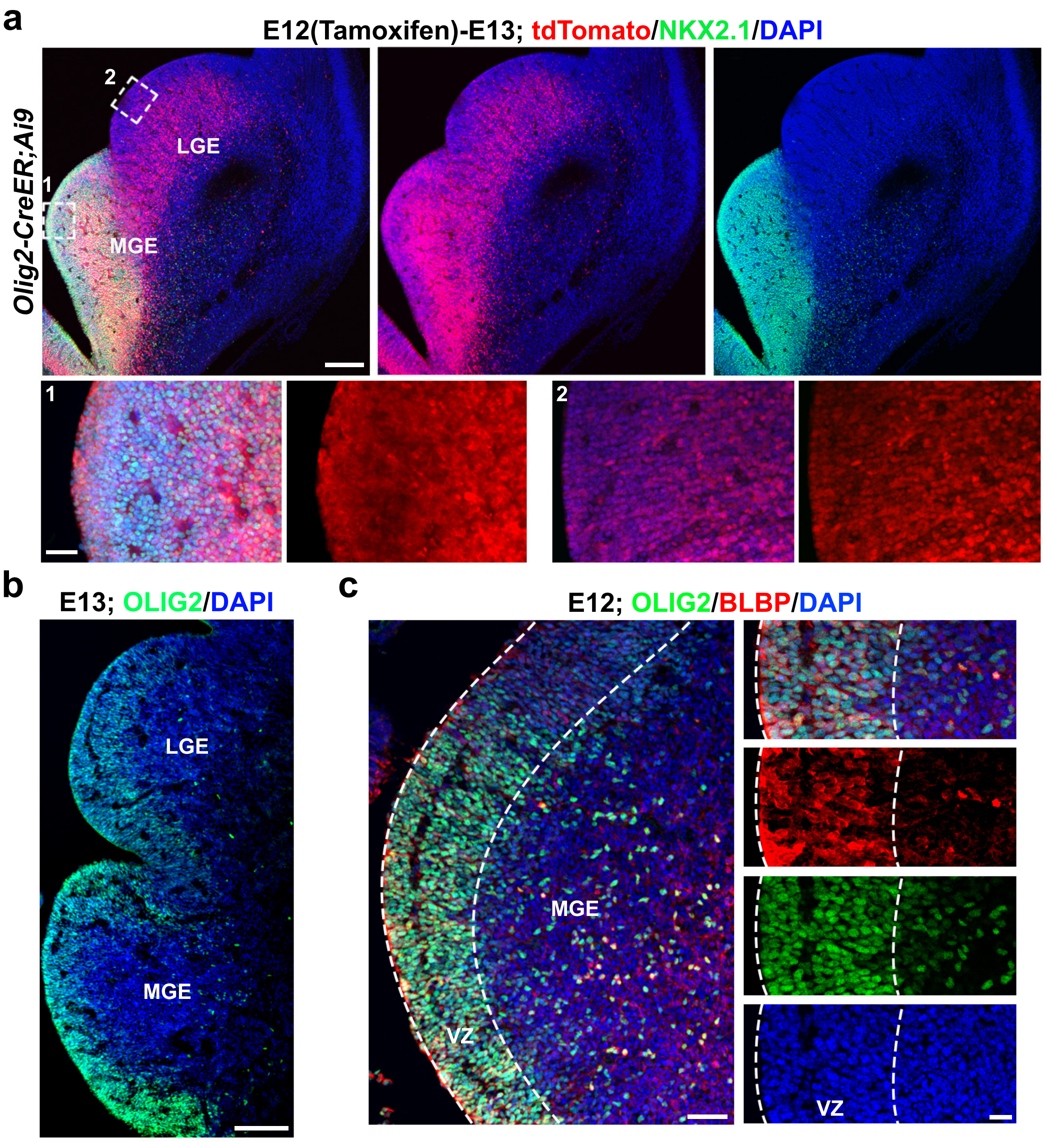

Sultan et al., Supplementary Figure 8

Supplementary Figure 8: OLIG2 is broadly expressed in RGPs in the MGE/PoA, as well as the LGE. (a) Images of an E13 *Olig2-CreER;Ai9* mouse brain, treated with tamoxifen at E12, and stained for tdTomato (red), NKX2.1 (green), and counterstained with DAPI (blue). High magnification images are shown at the bottom. TdTomato-expressing cells can be broadly observed in the VZ of the MGE, as well as the LGE. Scale bars: 150 μm and 30 μm. **(b)** Image of E13 MGE and LGE stained for OLIG2 (green) and counterstained with DAPI (blue). OLIG2-expressing cells can be found in the VZ of the MGE, as well as the LGE. **(c)** Images of E12 MGE stained for OLIG2 (green) and BLBP (red), and counterstained with DAPI (blue). High magnification images show densely packed OLIG2+ cells co-expressing BLBP, a bona-fide RGP marker, in the VZ. Broken lines indicate the VZ boundaries. Scale bars: 70 μm and 30 μm.

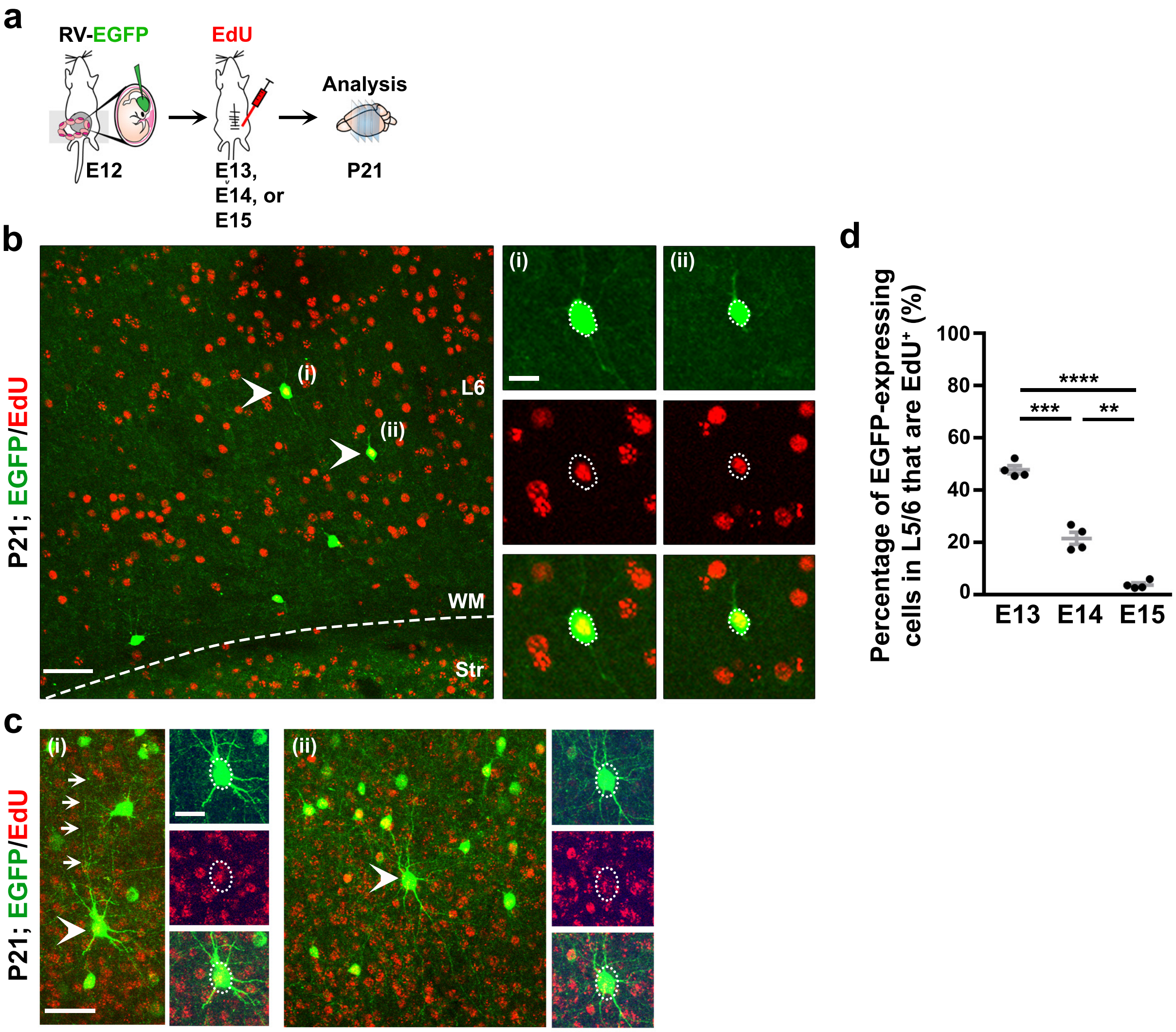

Sultan et al., Supplementary Figure 9

Supplementary Figure 9: Dividing NKX2.1$^+$ MGE/PoA RGPs produce predominantly non-chandelier cells at the early embryonic stage. (a) Schematic of the experimental design. **(b)** Image of P21 neocortex that received *in utero* injection of EGFP-expressing retrovirus at E12 followed by EdU injection at E13, stained for EGFP (green) and EdU (red). Arrow heads indicate EGFP-expressing interneurons labeled by EdU. The broken line indicates the boundary of the white matter (WM). High magnification images of EGFP-expressing interneurons in layer 6 labeled by EdU are shown to the right. Scale bar: 50 µm and 10 µm. **(c)** Representative images showing an EGFP-labeled Marinotti cell (i) and basket cell (ii) in L6 co-labeled with EdU. Arrowheads indicate cell body and small arrows indicate the L1-projecting axon of the labeled-Marinotti cell. High magnification images are shown to the right. Scale bars: 50 µm and 20 µm **(d)** Percentage of EGFP-expressing interneurons in layers 5/6 that are labeled by EdU. Grey lines represent mean ± SEM. Black dots represent individual hemispheres (n=4 per time point). *** P=0.0002 (E13-E14); **** P<0.0001 (E13-E15); ** P=0.002 (E14-E15) (Unpaired t-test with Welch's correction).

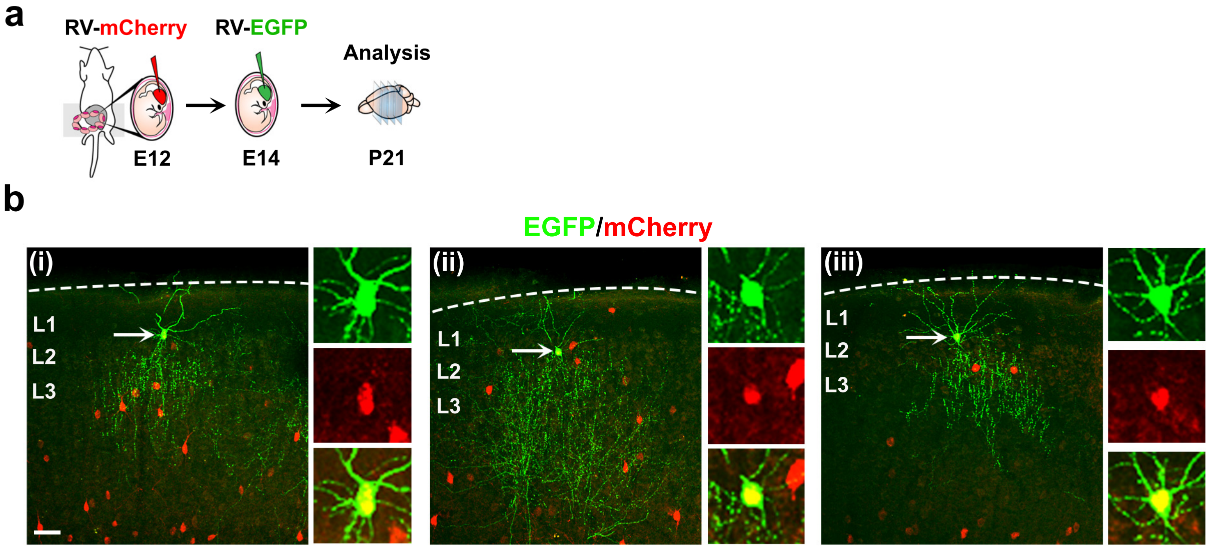

Sultan et al., Supplementary Figure 10

Supplementary Figure 10: Consecutive divisions of NKX2.1^{+} MGE/PoA RGPs generate superficial layer chandelier cells. (a) Schematic of the experimental design. **(b)** Representative images of P21 brains sequentially injected with mCherry- and EGFP- expressing retroviruses at E12 and E14, respectively, and stained with RFP (red) and EGFP (green). Arrows indicate superficial layer (L) chandelier cells expressing both mCherry and EGFP. High magnification images are shown to the right. Broken lines indicate the pial surface. Scale bar: 50 µm.

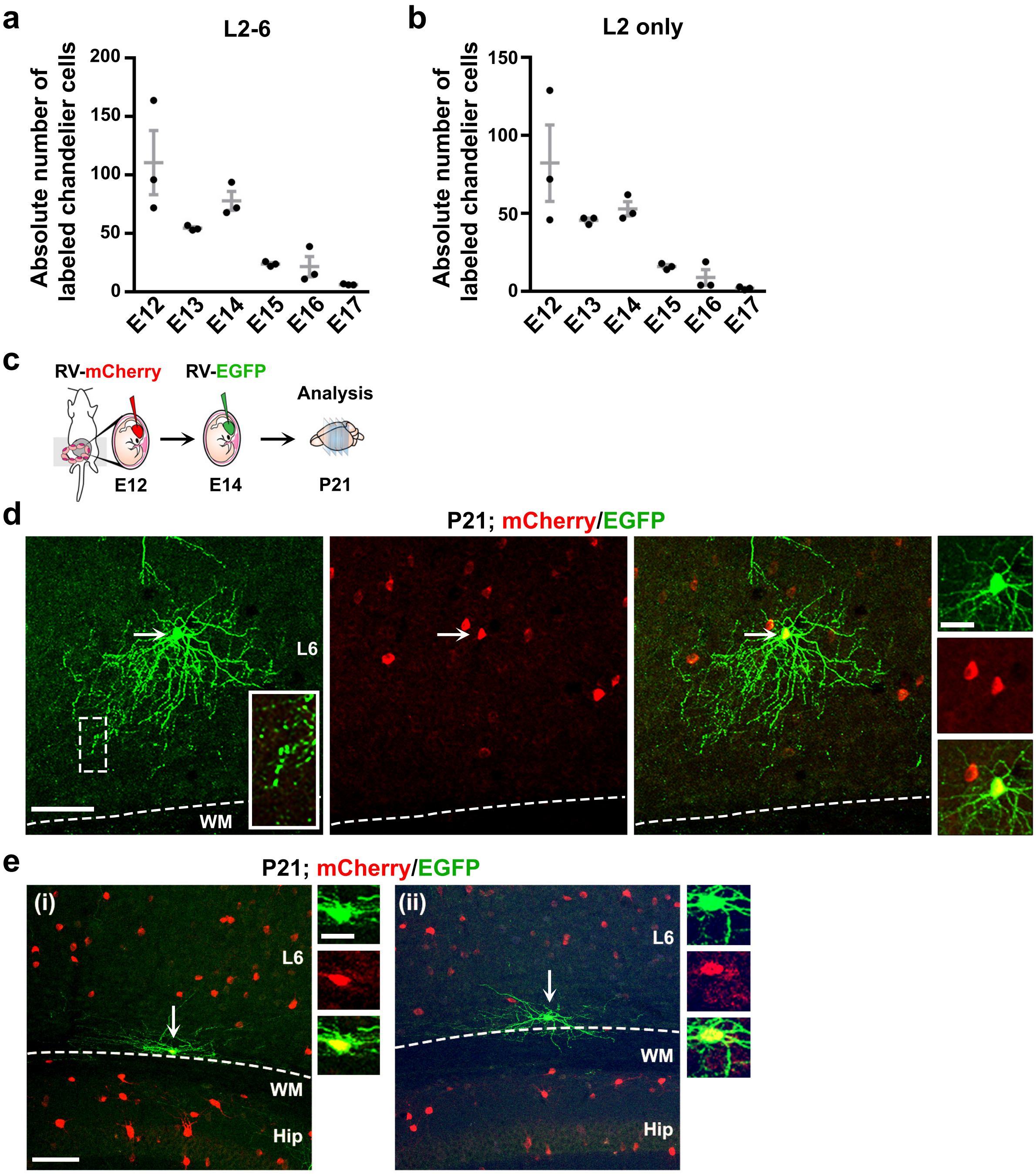

Sultan et al., Supplementary Figure 11

Supplementary Figure 11: Consecutive NKX2.1$^+$ MGE/PoA RGP divisions generate late-born deep layer chandelier cells. (a,b) Quantification of the absolute numbers of EGFP-labeled chandelier cells in layers (L) 2-6 (a) or L2 only (b) of the neocortex of P21 brains injected with EGFP-expressing retroviruses at E12-17. **(c)** Schematic of the experimental design. **(d)** Images of P21 neocortex of mice that received *in utero* injection of mCherry- and EGFP-retroviruses at E12 and E14, respectively, stained for mCherry (red) and EGFP (green). The arrows indicate a deep layer chandelier cell expressing both mCherry and EGFP. High magnification images of the vertical arrays of axonal cartridges characteristic of the chandelier cell (broken rectangles) are shown in the inset. High magnification images of the chandelier cell body are shown to the right. The broken line indicates the white matter (WM) boundary. Scale bars: 50 µm and 15 µm. (e) Respresentative images of layer 6 horizontal cells expressing both mCherry (red) and EGFP (green). High magnificaiton images are shown to the right. Broken lines indicate the white matter (WM) boundary. Scale bars: 50 µm and 10 µm.

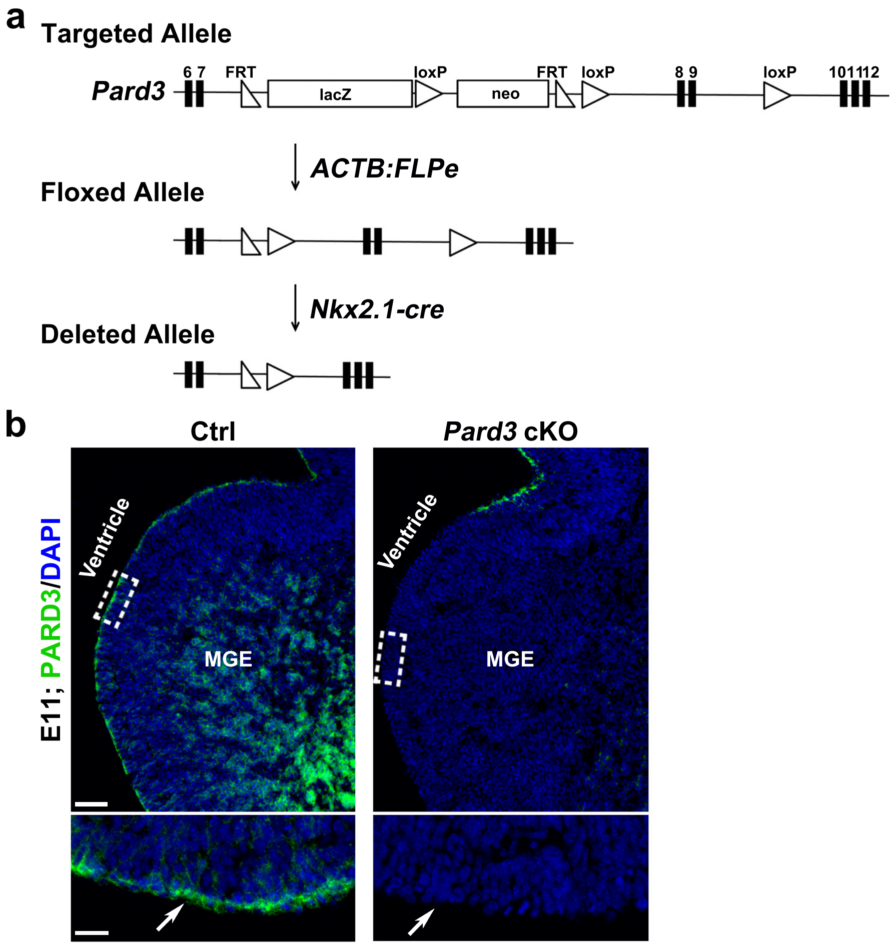

Sultan et al., Supplementary Figure 12

Supplementary Figure 12: Selective removal of PARD3 in NKX2.1^{+} MGE/PoA RGPs. (a) Schematic diagram showing the generation of *Nkx2.1-Cre;Pard3*$^{fl/fl}$ (referred to as *Pard3* cKO) mice. Numbered black boxes represent exons. Deletion of exons 8 and 9 leads to a frame shift of subsequent exons. **(b)** Images of control and *Pard3* cKO MGE at E11 stained for PARD3 (green) and counterstained with DAPI (blue). High magnification images of the VZ surface (broken rectangles) are shown at the bottom. Note the loss of PARD3 at the VZ surface in the *Pard3* cKO MGE (arrows). Scale bars: 70 µm and 15 µm.
