# Supplementary material for: Progressive divisions of multipotent neural progenitors generate late-born chandelier cells in the neocortex
Source: Nat Commun. 2018 Nov 2;9:4595. doi: 10.1038/s41467-018-07055-7 (PMC6214958; doi:10.1038/s41467-018-07055-7)
Supplement: Supplementary file 3 — Description of Additional Supplementary Files [file 41467_2018_7055_MOESM3_ESM.pdf]

Description of Additional Supplementary Files

File Name: **Supplementary Movie 1**
Description: **3D reconstruction of an E15 control brain hemisphere injected with EGFP-expressing retrovirus at E14.** The pial surface and the lateral ventricle are represented by colored lines; Green filled circles represent RGPs, yellow filled circles represent IPs, and red filled circles represent INs.

File Name: **Supplementary Movie 2**
Description: **3D reconstruction of an E15 *Pard3* cKO brain hemisphere injected with EGFP-expressing retrovirus at E14.** The pial surface and the lateral ventricle are represented by colored lines; Green filled circles represent RGPs, yellow filled circles represent IPs, and red filled circles represent INs.
